# Toward diffusion tensor imaging as a biomarker in neurodegenerative diseases: technical considerations to optimize recordings and data processing

**DOI:** 10.3389/fnhum.2024.1378896

**Published:** 2024-04-02

**Authors:** Hans-Peter Müller, Jan Kassubek

**Affiliations:** Department of Neurology, Ulm University, Ulm, Germany

**Keywords:** advances in computational neuroimaging for neurological diseases, magnetic resonance imaging, MRI, diffusion tensor imaging, DTI, neurodegenerative diseases, effect size, signal-to-noise ratio

## Abstract

Neuroimaging biomarkers have shown high potential to map the disease processes in the application to neurodegenerative diseases (NDD), e.g., diffusion tensor imaging (DTI). For DTI, the implementation of a standardized scanning and analysis cascade in clinical trials has potential to be further optimized. Over the last few years, various approaches to improve DTI applications to NDD have been developed. The core issue of this review was to address considerations and limitations of DTI in NDD: we discuss suggestions for improvements of DTI applications to NDD. Based on this technical approach, a set of recommendations was proposed for a standardized DTI scan protocol and an analysis cascade of DTI data pre-and postprocessing and statistical analysis. In summary, considering advantages and limitations of the DTI in NDD we suggest improvements for a standardized framework for a DTI-based protocol to be applied to future imaging studies in NDD, towards the goal to proceed to establish DTI as a biomarker in clinical trials in neurodegeneration.

## 1 Introduction

Magnetic resonance imaging (MRI) with diffusion tensor imaging (DTI) provides information on the microstructural processes of the central nervous system’s white matter (WM) *in vivo* (Pierpaoli and Basser, 1996). This technique has been used in different neurodegenerative diseases (NDD), e.g., in Alzheimer’s disease (AD) for both the early diagnosis and for monitoring disease progression (Oishi et al., 2011). Previous DTI studies in Parkinson’s disease (PD) have demonstrated alterations in multiple WM regions, particularly in the dopaminergic pathways (Zhang and Burock, 2020). DTI has shown microstructural abnormalities in patients with Huntington’s disease (HD) and is a tool to characterize how these abnormalities change with disease progression (Zhang et al., 2018). Furthermore, DTI plays a key role in cross-sectional and longitudinal imaging of WM alterations in motor neuron diseases (MND) like amyotrophic lateral sclerosis (ALS) (Basaia et al., 2019); cross-sectional DTI studies in patients with ALS identified the corticospinal tract as the major WM tract demonstrating microstructural alterations (Agosta et al., 2010; Kassubek et al., 2014; Müller et al., 2016; Braak et al., 2017).

Longitudinal DTI studies showed progression of WM degeneration, that way targeting a propagation-based biomarker in NDD (e.g., Gregory et al., 2015 – HD; Basaia et al., 2019 – MND; Kalra et al., 2020 – ALS; Torso et al., 2022 – AD; Hu Z. et al., 2023 – PD) and demonstrated the utility of DTI to monitor disease progression in NDD in a mono-or multicenter setting.

Based on these data, the aim was to reflect on suggestions for improvements of the scanning protocol as well as for the standardization of analysis (without focus on specific analysis software solutions) in order to provide a framework for future clinical trials regarding DTI scan protocol, analysis, and effect sizes.

## 2 Scanning protocol-related contributions to results of DTI studies in NDD and options of optimization

### 2.1 Analysis of microstructural alterations by DTI

As a basis for DTI data analysis, it has to be noted that WM tracts in the central nervous system consist of densely packed axons in addition to various types of neuroglia and other small populations of cells (Nieuwenhuys et al., 2019). The axonal membrane as well as the well-aligned protein fibers within an axon restrict water diffusion perpendicular to the fiber orientation, leading to anisotropic water diffusion in brain WM (Moseley et al., 1990). Myelin sheaths around the axons may also contribute to the anisotropy for both intra-and extracellular water (Mori and van Zijl, 2002). Diffusion anisotropy is mainly caused by the orientation of fiber tracts in WM and is influenced by its micro-and macrostructural features. Of the microstructural features (Basser et al., 1994; Mattiello et al., 1994), intraaxonal organization appears to be of greatest influence on diffusion anisotropy, besides the density of fiber and cell packing, degree of myelination, and individual fiber diameter. On a macroscopic scale, the variability in the orientation of all WM tracts in an imaging voxel influences its degree of anisotropy (Pierpaoli and Basser, 1996). DTI provides two types of information about the property of water diffusion: first, the orientation-independent extent of diffusion anisotropy (Pierpaoli and Basser, 1996) and second, the predominant direction of water diffusion in image voxels, i.e., the diffusion orientation (Pajevic and Pierpaoli, 1992). Since there are several challenges in displaying tensor data, the concept of diffusion ellipsoids has been proposed (Basser et al., 1994). The Eigendiffusivities of these ellipsoids represent the unidimensional diffusion coefficients in the main direction of diffusivities of the medium, i.e., the main axis of the ellipsoid represents the main diffusion direction in the voxel which coincides with the direction of the fibers, while the eccentricity of the ellipsoid provides information about the degree of anisotropy and its symmetry. Therefore, diffusion anisotropy metrics such as the fractional anisotropy (FA) could be defined for the parameterization of the voxel tensors (Le Bihan et al., 2001). Advanced techniques like Q-ball imaging (Tuch, 2004) may provide more sensitive white matter descriptors in single patients and lead to generalized measures of fractional anisotropy (Corbo et al., 2014). Thus, the clinical role of DTI in various disease processes, especially NDDs, is emerging (Tae et al., 2018).

These DTI-based metrics have shown to map age-related alterations over the life span in the human brain; such age-dependent changes also seem to exhibit regional differences with respect to the brain anatomy (Salat et al., 2005; Westlye et al., 2010). Furthermore, a regional tract-specific age dependency which requires also non-linear corrections for different age ranges has been demonstrated (Behler et al., 2021). Based on these findings, DTI also acts as a cross-validation technique for age-dependent cerebral blood flow alterations in the human brain (Damestani et al., 2024).

### 2.2 DTI protocol

Standardized DTI protocols will, at least currently, be performed at 1.5 T or 3.0 T clinical scanners, because scanners with higher magnetic field (ultrahigh field scanners) would provide better signal-to-noise ratio but are still rare and hardly available for multicenter trials. The protocols include full-brain coverage using a 2-D echo planar imaging sequence; about axial 70 slices, slice thickness 2.0 mm, voxel size 2.0 × 2.0 × 2.0 mm^3^, field of view 256 × 256 mm^2^, matrix 128 × 128, five b_0_ images, and 30 or more diffusion gradient directions with a b value of 1,000 s/mm^2^. The recording of more than one b_0_ image could help to improve the signal-to-noise-ratio (SNR). Good examples of standardized prospective multicenter protocols are provided in (Hobbs et al., 2015 – HD; Nir et al., 2015 – AD; Kalra et al., 2020 – ALS). However, a major drawback of DTI is that it assumes a single water pool with Gaussian diffusion in each voxel and does not account for structural heterogeneity which reduces the specificity of the derived indices (Alexander et al., 2001; Basser and Jones, 2002), neurite orientation dispersion and density imaging (NODDI) is a multishell diffusion technique (Zhang et al., 2012) that assumes further types of microstructural environments and could easily be added to a standardized DTI protocol, if *b*-values of 2000 s/mm^2^ are available.

For the application of fiber tracking (Mori and van Zijl, 2002), differently oriented fiber bundles inside one voxel are incorrectly modeled by a single tensor. High Angular Resolution Diffusion Imaging (HARDI) aims at using more complex models, such as a two-tensor model, for estimating two fiber bundles (Caan et al., 2007). Although standard DTI is an established default tool, acquisition with stronger diffusion weightings beyond the DTI regimen is now possible by NODDI, that way enabling even more detailed characterization of tissue microstructures, e.g., in the early diagnosis of AD (Takahashi et al., 2024) or tau deposition in AD (Weston et al., 2023).

The schedule of visits could be optimized taking possible costs and patient burden into account. Repeated DTI scans during one given subject’s visit could significantly improve the effect size (Behler et al., 2022a). Thus, it is recommended to increase the number of DTI scan repetitions at one visit. Note that the scans should be independent, i.e., subject repositioning in the scanner is recommended.

For longitudinal studies in NDD with a rapidly progressive disease course, e.g., ALS, one baseline and two follow-up visits were advised over a total observation period of about 12 months (time interval between baseline and follow-up 2) to monitor the course of disease (e.g., Cardenas-Blanco et al., 2016; Kassubek et al., 2018; Kalra et al., 2020). For longitudinal studies in NDD with a slowly progressive disease course, multiple follow-up scans are possible and could even be spread over several years, e.g., in PD with one baseline and two follow-up visits 6 years apart (Shih et al., 2023).

Independent of the progression rate, it has to be noted that, if one baseline and two follow-up scans are recorded, the first follow-up visit is suggested to balance observing change and minimizing attrition so that the individual schedule should be such that the timing of follow-up 1 does not bisect the entire observation period between baseline and follow-up two. The unfavorable choice of a symmetrical bisectioning of the observation interval was demonstrated in a cohort of HD patients with one baseline and 2 follow-up scans (Müller et al., 2021). The shortcoming with splitting the observation time into two identical periods is that regression models, due to their intrinsic properties, underweight follow-up visit 1, with the result that the progression slope is mainly determined by baseline and follow-up 2.

### 2.3 Quality control

In addition to common MRI artifacts (truncation, aliasing, chemical shift, banding, pile-up, blurring, spikes, etc.) (Krupa and Bekiesińska-Figatowska, 2015), there are specific challenges that one may encounter when using MRI scanner gradient hardware for diffusion MRI, especially in terms of eddy currents and sensitivity to motion (Le Bihan et al., 2006). According to an established quality control protocol (Dubois et al., 2014; Müller et al., 2014), corrupted gradient directions as well as motion artifacts could be excluded and corrupted slices or volumes could be resampled from further analysis prior to correction of eddy current-induced geometric distortions (Shen et al., 2004). In particular, if the analysis focuses on one parameter like FA, the omission of single GD has only little effect on the result and thus the omission of corrupt GD allows a more precise determination of the resulting values.

### 2.4 Subject samples

#### 2.4.1 Effect size and sample size calculations

The effect size is estimated by Cohen’s *d*, i.e.,


(1) $$d=\frac{\mu_{NDD}-\mu_{\mathrm{controls}}}{\sigma_{NDD,\mathrm{controls}}}$$

with


$$\sigma_{NDD,\mathrm{controls}}^{2}=\frac{\left(N_{NDD}-1\right)\sigma_{NDD}^{2}+\left(N_{\mathrm{controls}}-1\right)\sigma_{\mathrm{controls}}^{2}}{N_{NDD}+N_{\mathrm{controls}}-2}$$

Sample size calculations usually were based on a two-sided significance level (*α*) of *5%* and a power (*1-β*) of *80%*. Then, the sample size n could be calculated by Kadam and Bhalerao (2010)


(2) $$n=2{\left(Z_{a}+Z_{1-\beta }\right)}^{2}/d^{2}$$

and with *Z_a_ = 1.96* and *Z_1-β_ = 0.84*, the sample size could be approximated to


(3) $$n=15.7/d^{2}$$

Effect size and sample size calculations for cross-sectional comparisons yield an estimation of baseline effects in a clinical study, whereas estimates for longitudinal comparisons yield effect sizes and sample sizes required to observe time-dependent changes.

To obtain maximum effect sizes [high values of *d* (Eq. 1)] in clinical trials, high differences between the mean values (of any quantity) of NDD patients and controls should be obtained. It is of high relevance, however, to obtain minimum standard deviations [σ (Eq.1)]. This minimization is discussed in the following.

#### 2.4.2 Selection of subject samples for clinical trials

In order to be able to identify a disease-specific alteration (in a given parameter), an optimized and representative control sample should be used. Both inter-subject variability, scanner and environmental noise as well as subject motion or *ad hoc* scan specific noise contribute to the DTI signal (Müller et al., 2014).

The averaged recorded signal of a group of *N* subjects (each with one scan) can be split into (Note: in the following, N>>1 is assumed)


(4) μ=F+L+ΔG

with.

$\mu =\frac{1}{N}\sum_{i}^{N}S_{i};\;\sigma^{2}=\frac{1}{N}\sum_{i}^{N}{\left(S_{i}-\mu \right)}^{2}$ *S_i_* are the individual measured signals for the respective subject.

$L=\frac{1}{N}\sum_{i}^{N}L_{i}$ ; *L_i_* are the individual system noise values for a single scan including partial volume effects due to different subject positioning.

$\Delta G=\frac{1}{N}\sum_{i}^{N}\Delta G_{i}$ ; Δ*G_i_* are the individual disease related alterations for the respective subject; Δ*G_i_* = 0 for controls; for NDD patients Δ*G_i_* are assumed to be considerably larger than the age-related alterations.

$F=\frac{1}{N}\sum_{i}^{N}F_{i}$ ; *F_i_* are the individual values of a parameter for the respective subject.

*F_i_* can be split into a mean value (representing the “normal” value of a parameter) and the individual variability *ΔF_i_*


(5) $$F=\bar{F}+\frac{1}{N}\sum_{i}^{N}\Delta F_{i}$$

$\bar{F}+\Delta F_{i}=F_{i}$ are the individual values of a parameter for the respective subject.

Now, the effect size of a two sample (NDD patients and controls) study is


(6) $$d_{\mathrm{cross}}=\frac{\left(F_{NDD}+L_{NDD}+\Delta G\right)-\left(F_{\mathrm{controls}}+L_{\mathrm{controls}}\right)}{\left(\sigma_{NDD,\mathrm{controls}}\right)}$$

$${\left(\sigma_{NDD,\mathrm{controls}}\right)}^{2}=\frac{N_{NDD}{\left(\sigma_{NDD}\right)}^{2}+\;N_{\mathrm{controls}}{\left(\sigma_{\mathrm{controls}}\right)}^{2}}{N_{NDD}+N_{\mathrm{controls}}}$$

Thus,


(7) $$d_{\mathrm{cross}}=\frac{\Delta G-\left(\frac{1}{N_{NDD}}\sum_{i}^{N_{NDD}}\left(\Delta F_{i}+L_{i}\right)+\frac{1}{N_{\mathrm{controls}}}\sum_{i}^{N_{\mathrm{controls}}}\left(\Delta F_{i}+L_{i}\right)\right)}{\left(\sigma_{NDD,\mathrm{controls}}\right)}$$

That way the noise and inter-individual variability are contained $\left(N_{\mathrm{controls}}+N_{NDD}\right)$ times.

Coherent signal averaging can be expressed by Rompelman and Ros (1986)

(8) $$\begin{array}{c} S=\frac{1}{N}\sum_{i}^{N}S_{i}=\frac{1}{N}\sum_{i}^{N}F_{i}+\frac{1}{N}\sum_{i}^{N}L_{i}\\ =F+\frac{1}{N}\sum_{i}^{N}L_{i}=\bar{F}+\frac{1}{N}\sum_{i}^{N}\left(\Delta F_{i}+L_{i}\right) \end{array}$$

$SNR=\frac{N\bar{F}}{\sum_{i}^{N}\left(\Delta F_{i}+L_{i}\right)}=\frac{N\bar{F}}{\sqrt{N}\sigma_{N}}∼\sqrt{N}$

with a root mean square value of the noise of *σ_N_*, i.e., after *N* averages (normally distributed values), the SNR scales as


(9) $$SNR_{N}=\sqrt{N}SNR$$

**Figure 1:**
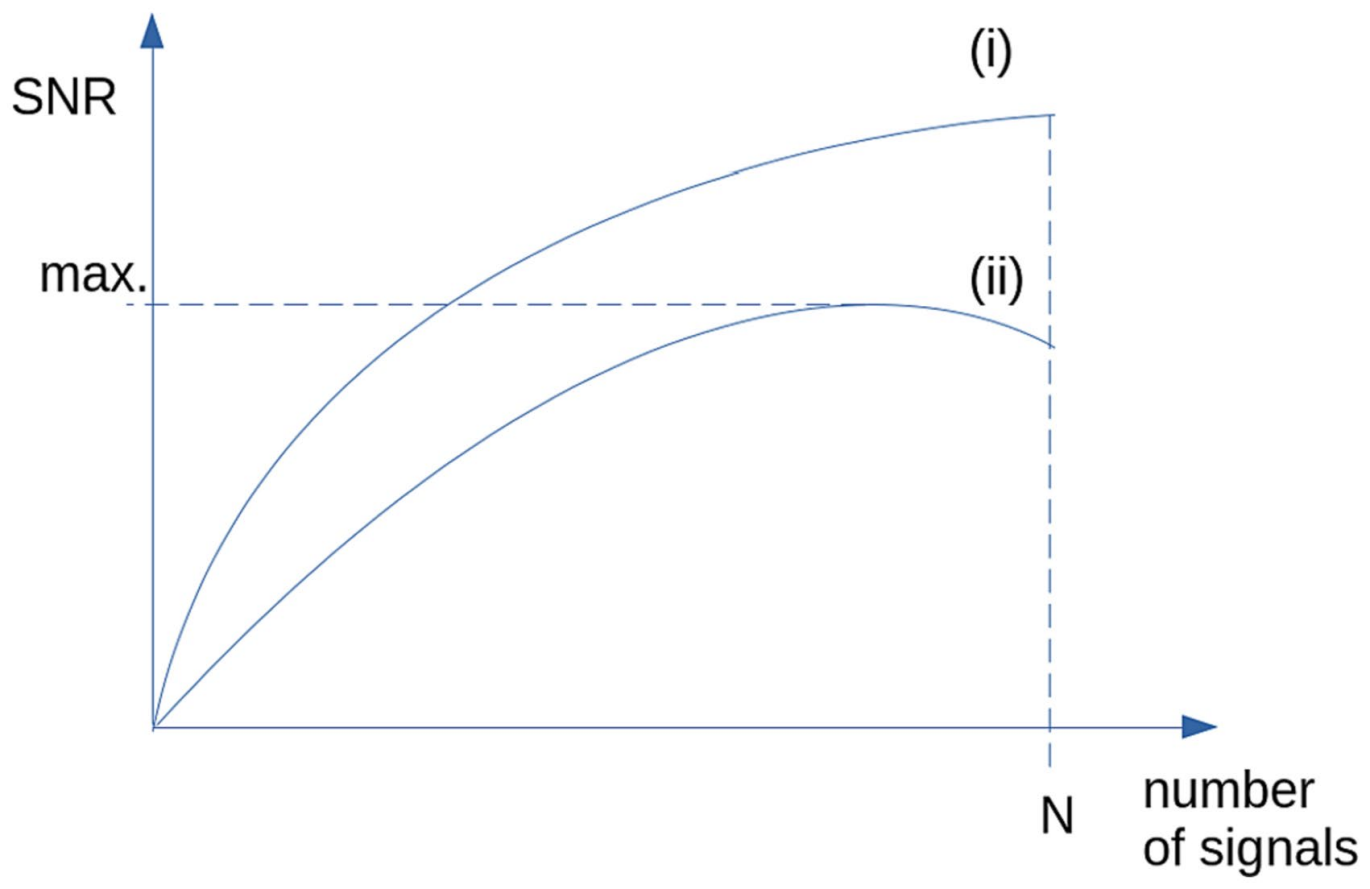
Schematic illustration of the SNR: (i) SNR is growing with N for the case of identical signals and stationary, normally distributed noise. (ii) For non-stationary noise (i.e., high inter-subject variability), SNR shows a maximum for selected signals (e.g., by categorized clustering).

Based on studies in a completely different field of research, that is the optimization of averaged heartbeats, there are techniques that allow an optimized representative data collective to be selected from a larger data collective of controls (Mühler and von Specht, 1999; DiPietroPaolo et al., 2005). That way, the variability of any parameter from the control sample could be reduced. This reduction of variability includes inter-subject variability as well as scanner and environmental noise. The simplest technique is to use median and two-sided 80% percentile, more effective is the use of a categorized clustering (DiPietroPaolo et al., 2005) (schematic illustration in Figure 1) to obtain a maximum SNR.

When applying the percentile criterion or a categorized clustering approach, Eq. 5 is replaced by


(10) $$F_{M}=\bar{F}+\frac{1}{pN}\sum_{i}^{pN}\Delta F_{i}$$

where *p* is a value between 0 and 1, representing the amount of averaged data. Then, also the system noise is altered:


(11) $$L_{M}=\frac{1}{pN}\sum_{i}^{pN}L_{i}$$

That way, as the mean value of the controls is approximately stable and the standard deviation of controls is reduced, higher effect sizes could be obtained. Thus, the following recommendation can be given: in case of a high variability in the controls` data (also caused by outliers), the appropriate reduction of the control sample toward a more representative one leads to an increase in the effect size. In a given study, the careful selection of a representative controls sample could be more effective in terms of effect size optimization than merely increasing the number of controls by an unselective inclusion into the study.

The same considerations apply to the patient sample, with a high grade of inter-individual variability particularly in brain regions that are affected at variable degrees by the given NDD. Nevertheless, attention should be directed to careful patient selection based on the research question, also excluding patients with additional medical conditions if necessary.

That way, the noise and inter-individual variability are contained $\left(pN_{\mathrm{controls}}+qN_{NDD}\right)$ times ( 0<p,q<1 ) and the effect size (Eq. 7) is increased. Furthermore, $\sigma_{NDD,\mathrm{controls}}$ (Eq. 7) is also reduced, that way increasing the effect size.

#### 2.4.3 What can we gain from longitudinal scans of controls?

Longitudinal scans of controls (when age-related alterations can be neglected, e.g., scans within time intervals in the range of months) mainly provide the following information:

- estimation of system noise (Eq. 4).
- estimation of inter-individual variability.
- longitudinal alterations of controls (at the group level) – these should be negligible over such a short period.

Longitudinal alterations in controls should have no impact on the further analysis. The comparison of longitudinal alterations in patients and in controls is therefore dominated by system noise (i) and inter-individual variability (ii). Therefore, it is recommended that efforts should be spent in accumulating a high amount of different controls at baseline (rather than longitudinal scans of controls), both to increase the SNR and to decrease the variability in signals of controls.

As a consequence, system noise (i) and inter-individual variability (ii) together could be used at the group level to act as a “detection threshold,” i.e., only longitudinal alterations in patients that exceed this threshold are defined as “detectable.”

## 3 Postprocessing-related contributions to results of DTI studies in NDD: options of optimization

### 3.1 DTI data analysis

Considering the effect of experimental noise on DTI metrics (Bastin et al., 1998; Seo et al., 2019), statistical analysis is provided by several software packages [e.g., Statistical Parametric Mapping (SPM) (Penny et al., 2006), FMRIB Software Library (FSL) (Jenkinson et al., 2012), Tensor Imaging and Fiber Tracking (TIFT) (Müller et al., 2007)]. Analysis could be performed as unbiased whole brain-based voxelwise comparison of DTI metrics (Müller et al., 2014), or as hypothesis-guided region of interest (ROI) or tract of interest (TOI) analyses [tract-based spatial statistics (TBSS) (Smith et al., 2006) or tractwise fractional anisotropy statistics (TFAS) (Mueller et al., 2007)]. Analyses pipelines combining several toolboxes have also been introduced (e.g., Cui et al., 2013; Soares et al., 2013).

The principles of microstructural analysis by DTI have been taken use of in many mono-and multicenter studies in NDD:

‣ AD (e.g., Nir et al., 2015 – multicenter (FSL, TBSS); Douaud et al., 2011; Bourbon-Teles et al., 2023; Zhang and Zhan, 2023 – monocenter (TBSS); Chen Y. et al., 2023 – review; Takahashi et al., 2024 – NODDI).
‣ ALS [e.g., Canu et al., 2011 – monocenter (in house); Corbo et al., 2014 (TBSS); Basaia et al., 2019 – review; Kalra et al., 2020 – multicenter (TIFT); Turner et al., 2011; Filippi et al., 2015].
‣ HD [e.g., Douaud et al., 2009; Gregory et al., 2015; Zhang J. et al., 2018 – multicenter (in house, FSL)].
‣ PD [e.g., Gorges et al., 2019 – multicenter (*TIFT*); Zhang and Burock, 2020 – review; Shih et al., 2023 – monocenter (FSL)].

The cross-sectional comparison of NDD patient groups to healthy controls aims at the definition of specific brain regions and the related patterns of alterations. Beyond this initial cross-sectional research in specific NDDs, a “pseudo-longitudinal” analysis of DTI could be performed: cross-sectional results could also be further analyzed by taking the disease status of the individuals as time-axis in order to map increasing alterations or spreading patterns during the course of ALS (Kassubek et al., 2014; Müller et al., 2023) or association patterns similar to the neuropathological Braak staging of AD (Wen et al., 2021) could be analyzed. Meta-analyses of DTI metrics in PD contribute to increasing the knowledge of PD pathophysiology by addressing the possibility of follow-up of the disease severity and associated brain structural modulations using *in vivo* imaging (Atkinson-Clement et al., 2017).

### 3.2 Statistical analysis

In case of unequal sample sizes, the Welch-test should be used which is an adaption of Student’s t-test and is more reliable when the two samples have unequal variances. Welch’s *t*-test defines the statistic by


(12) $$t=\frac{\mu_{NDD}-\mu_{\mathrm{controls}}}{\sigma_{NDD,\mathrm{con}t\mathrm{rols}}}$$

with $\sigma_{NDD,\mathrm{controls}}^{2}=\sigma_{NDD}^{2}/N_{NDD}+\sigma_{\mathrm{controls}}^{2}/N_{\mathrm{controls}}$

Prior to statistical analysis, input data should be tested for normal distribution. In case of not normally distributed data, the samples should first be checked with regard to their distribution and the contributing data (see subsection *“Selection of subject samples for clinical trials”*) and, if not normally distributed, non-parametric testing, e.g., Mann–Whitney *U*-test, should be applied.

### 3.3 Advantage of comparisons of longitudinal data with a control data set at baseline

Previous studies that analyzed longitudinal alterations in ALS patients (Zhang et al., 2016; Kassubek et al., 2018; Kalra et al., 2020; Shih et al., 2023) calculated the effect size of longitudinal differences in a given DTI metric by


(13) $$\begin{array}{c} d_{\mathrm{long},1}=\frac{\Delta \mu_{NDD}-\Delta \mu_{\mathrm{controls}}}{\left(\Delta \sigma_{NDD,\mathrm{controls}}\right)}\\ =\frac{\frac{1}{N_{NDD}}\sum_{i}^{N_{NDD}}\left({\left(S_{i,FUP}-S_{i,BL}\right)}_{NDD}\right)-\frac{1}{N_{\mathrm{controls}}}\sum_{i}^{N_{\mathrm{controls}}}\left({\left(S_{i,FUP}-S_{i,BL}\right)}_{\mathrm{control}s}\right)}{\left(\Delta \sigma_{NDD,\mathrm{controls}}\right)} \end{array}$$

with


$${\left(\Delta \sigma_{NDD,\mathrm{controls}}\right)}^{2}=\frac{N_{NDD}{\left(\Delta \sigma_{NDD}\right)}^{2}+\;N_{\mathrm{controls}}{\left(\Delta \sigma_{\mathrm{controls}}\right)}^{2}}{N_{NDD}+N_{\mathrm{controls}}}$$

$$\Delta \mu_{\mathrm{group}}=\mu_{\mathrm{group},FUP}-\mu_{\mathrm{group},BL}\text{,}$$

${\left(\Delta \sigma_{\mathrm{group}}\right)}^{2}=\frac{1}{N}\sum_{i}^{N}{\left({\left(S_{i,FUP}-S_{i,BL}\right)}_{\mathrm{group}}-\Delta \mu_{\mathrm{group}}\right)}^{2}$


group∈NDD,controls

$\Delta \mu_{NDD},\Delta \mu_{\mathrm{controls}}$ are the average longitudinal alterations in controls and in NDD patients at the group level. Note: in this approach a longitudinal time interval normalization is necessary (Kassubek et al., 2018).

Eq. 13 can be written as


(14) $$d_{\mathrm{long},1}=\frac{\left(\begin{array}{l} \left(F_{NDD,FUP}+L_{NDD,FUP}+\Delta G_{FUP}-\;F_{NDD,BL}-L_{NDD,BL}-\Delta G_{BL}\right)\\ -\;\left(F_{\mathrm{controls},FUP}+L_{\mathrm{controls},FUP}-\;F_{con\mathrm{trols},BL}-L_{\mathrm{controls},BL}\right) \end{array}\right)}{\left(\Delta \sigma_{NDD,\mathrm{controls}}\right)}$$

Now, the effect size of a two sample (NDD patients and controls) study is


(15) $$\begin{array}{l} d_{\mathrm{long},1}=\\ \frac{\left(\Delta G_{FUP}-\Delta G_{BL}\right)-\left(\frac{1}{2N_{NDD}}\sum_{i}^{2N_{NDD}}\left(\Delta F_{i}+L_{i}\right)+\;\frac{1}{2N_{\mathrm{controls}}}\sum_{i}^{2N_{\mathrm{control}s}}\left(\Delta F_{i}+L_{i}\right)\right)}{\left(\Delta \sigma_{NDD,\mathrm{controls}}\right)} \end{array}$$

That way the noise and inter-individual variability are contributing $\left(2N_{\mathrm{controls}}+2N_{NDD}\right)\;\mathrm{times}\text{.}$

This approach also takes longitudinal alterations in controls into account, that way, mainly incorporating additional noise components due the information input of longitudinal scans of controls. Since the target is the detection of alterations in NDD patients (see subsection “*What can we gain from longitudinal scans of controls?”*), the alterations of inter-subject variability of controls $\Delta F_{i}$ (as obtained from longitudinal scans of controls) is off target and would only lead to an additional noise component in the results.

An alternative approach (e.g., Cardenas-Blanco et al., 2016; Loane et al., 2016) is to analyze data without follow-up control visits; then, Eq. 15 simplifies to


(16) $$d_{\mathrm{long},2}=\frac{\Delta \mu_{NDD}}{\Delta \sigma_{NDD}}=\frac{\left(\Delta G_{FUP}-\Delta G_{BL}\right)-\;\left(\frac{1}{2N_{NDD}}\sum_{i}^{2N_{NDD}}\left(\Delta F_{i}+L_{i}\right)\right)}{\left(\Delta \sigma_{NDD}\right)}$$

Then the noise and inter-individual variability are contained $2N_{NDD}$ times. This approach (ideally with a large control sample at baseline) shows a higher impact on the effect size as ${\left(\Delta \sigma_{\mathrm{controls}}\right)}^{2}=0$ .Thus


(17) $$d_{\mathrm{long},2}>d_{\mathrm{long},1}$$

However, this approach without follow-up of the controls is particularly suitable for cross-sectional comparisons of each visit to a (large) database of controls (Eq. 7) so that optimal effect size can be obtained at any visit. This is particularly suitable for a 3-D visualization of longitudinal alterations (unbiased whole brain-based voxelwise analysis) or for the display of longitudinal changes in defined structures (e.g., Cardenas-Blanco et al., 2016). That means that higher effect sizes could be gained when data of each visit (of NDD patients) are compared to a (large and optimized) control sample at baseline.

### 3.4 Multiparametric statistics and application of artificial intelligence

Multiparametric or multimodal imaging has a high impact on the quality and reliability of results. The investigation of the relationship between WM alterations with further factors (atrophy, vascular disease, or, e.g., amyloid burden) and clinical features increases the set of analyzed parameters and thus offers possibilities to apply methods of multiparametric analyses, e.g., in AD and Lewy body dementia (Donaghy et al., 2020). Recent multiparametric analyses showed no association between multiple biomarkers of cerebral microvascular function and WM connectivity (using DTI to quantify the number and organization of WM connections) in a large cohort of some thousand participants (Beran et al., 2024), however, further MRI techniques to assess blood–brain-barrier permeability or cerebrovascular reactivity and microvascular perfusion at the tissue level could be combined with WM connectivity analysis of DTI (Beran et al., 2024). Multimodal association analyses of DTI with amyloid beta and tau positron emission tomography (PET) show potential to provide information relating to underlying tau deposition in AD (Weston et al., 2023).

DTI can be used to tract-wise map correlates of the sequential disease progression and, therefore, to assess disease stages, e.g., in ALS (Kassubek et al., 2014, 2018). A technical improvement in reliability of the analysis was reached by applying a multistage classifier based on Bayesian statistics (Behler et al., 2022b) with the significant advantage of Bayesian statistics for multimodal issues by incorporating prior knowledge about the patient into the algorithm.

A multiparametric set of variables (including the predictive value of microstructural integrity) could be used for association with clinical phenotype features like cognitive performance (Power et al., 2019), the improvement of diagnostic accuracy (Piersson et al., 2021), or to assess the predictive value on survival (Agosta et al., 2019; Kuan et al., 2023) in NDD.

The predictive value of microstructural integrity has been used by artificial intelligence (AI) as machine learning (ML) tools; especially convolutional neural networks (CNN), support vector machines (SVM), and random Forrest models (RFM) successfully contributed to neuroimaging studies in NDDs.

#### 3.4.1 Convolutional neural networks

Convolutional neural networks consists of neurons in different layers, where the restriction of a set is defined by the number of neurons in the output layer. The restriction is primarily based on the weights and connections of individual neurons and their interdependencies (Le Cun et al., 2015). Neurons are essentially functions, often similar to XOR or functions and have a weight that can be interpreted as a threshold potential to be crossed by the input values.

Multi-Kernel CNN can accurately identify AD and mild cognitive impairment (MCI) features from DTI data, and the generated fiber probability map can represent the risk status of AD and MCI (Deng et al., 2023). A two-layer stacking ensemble learning framework with fusing multimodal features has been developed for accurately identifying early PD from healthy controls by combining several AI algorithms. This model performed an accuracy of 96.88%, a precision of 100% (Yang et al., 2021a).

#### 3.4.2 Support vector machines

In SVMs, a set of parameters is divided into separate groups by a hyperplane (Noble, 2006). This hyperplane is generated by vectors, derived by differentiating parameters associated with a clinical target. The hyperplane is optimized as a decision boundary in a multidimensional space through an AI training process.

Discrimination of NDD patients from controls could be performed by the application of an SVM based on ROI-or TOI-based FA values of previously defined NDD-associated brain structures (AD – Zhang et al., 2011; Houria et al., 2022; PD – Haller et al., 2012; Huang et al., 2023; ALS – Chen et al., 2020; Kocar et al., 2021; Münch et al., 2022).

#### 3.4.3 Random Forrest models

The core of the RFM is the creation of multiple decision trees during the training phase. Each tree is constructed using a random subset of the training data and features, following the principle of bootstrapping (Breiman, 2001); the algorithm incorporates randomness in two primary aspects:

(i) Data Sampling (Bootstrap Aggregating or Bagging): Each decision tree is trained on a randomly sampled subset of the data, known as a bootstrap sample. This sampling ensures diversity among the trees. (ii) Feature Selection: At each split in a tree, a random subset of features is considered. This randomness in feature helps to reduce variance between trees, thereby increasing the overall robustness of the model.

Successful applications of RFM to DTI data from NDD patients were performed in AD (Yang J. et al., 2022), PD (Chen B. et al., 2023), and ALS (Sarica et al., 2017), each discriminating NDD from controls with high accuracy.

### 3.5 Harmonization of data in multicenter trials

Especially in multicenter trials, different factors may contribute to the variability of DTI data of controls and NDD patients. Although the precise influence of each source of variation could not be delineated, investigating group FA differences between patients and controls on systematic between-center differences should be investigated prior to pooling across centers. Furthermore, center-specific sources of variability on DTI metrics, e.g., scanner-specific variability, environmental noise and specific factors such as scanning time, might be present in single center studies but will only slightly influence comparisons at the group level (Müller et al., 2013). Although multicenter studies could allow a direct merging of DTI metrics across centers (Müller et al., 2013), especially in case of different scan protocols, merging of DTI metrics requires the application of a strategy to regress out confounders such as field strength, echo time, or number of gradient directions (Müller et al., 2016). Especially, FA is influenced by the voxelsize (Oouchi et al., 2007) and thus needs harmonization in case of different scan protocols in different centers. Harmonization could be performed by 3-D voxelwise linear correction matrices (Rosskopf et al., 2015); recently, a post-processing technique based on rotation invariant spherical harmonics features was introduced to mitigate cross-scanner differences in DTI metrics (De Luca et al., 2022).

The development of a Domain Shift Analyzer for MRI (DSMRI) which was designed explicitly for multicenter MRI datasets (Kushol et al., 2023a,b) allows for the identification of NDD as demonstrated for ALS cases (Kushol et al., 2023c), thus offering the possibility to investigate neuroimaging modalities like functional MRI (fMRI) and DTI within a similar environment.

## 4 Discussion

### 4.1 Summary of recommendations for a standardized DTI scan protocol and DTI analysis

**Figure 2:**
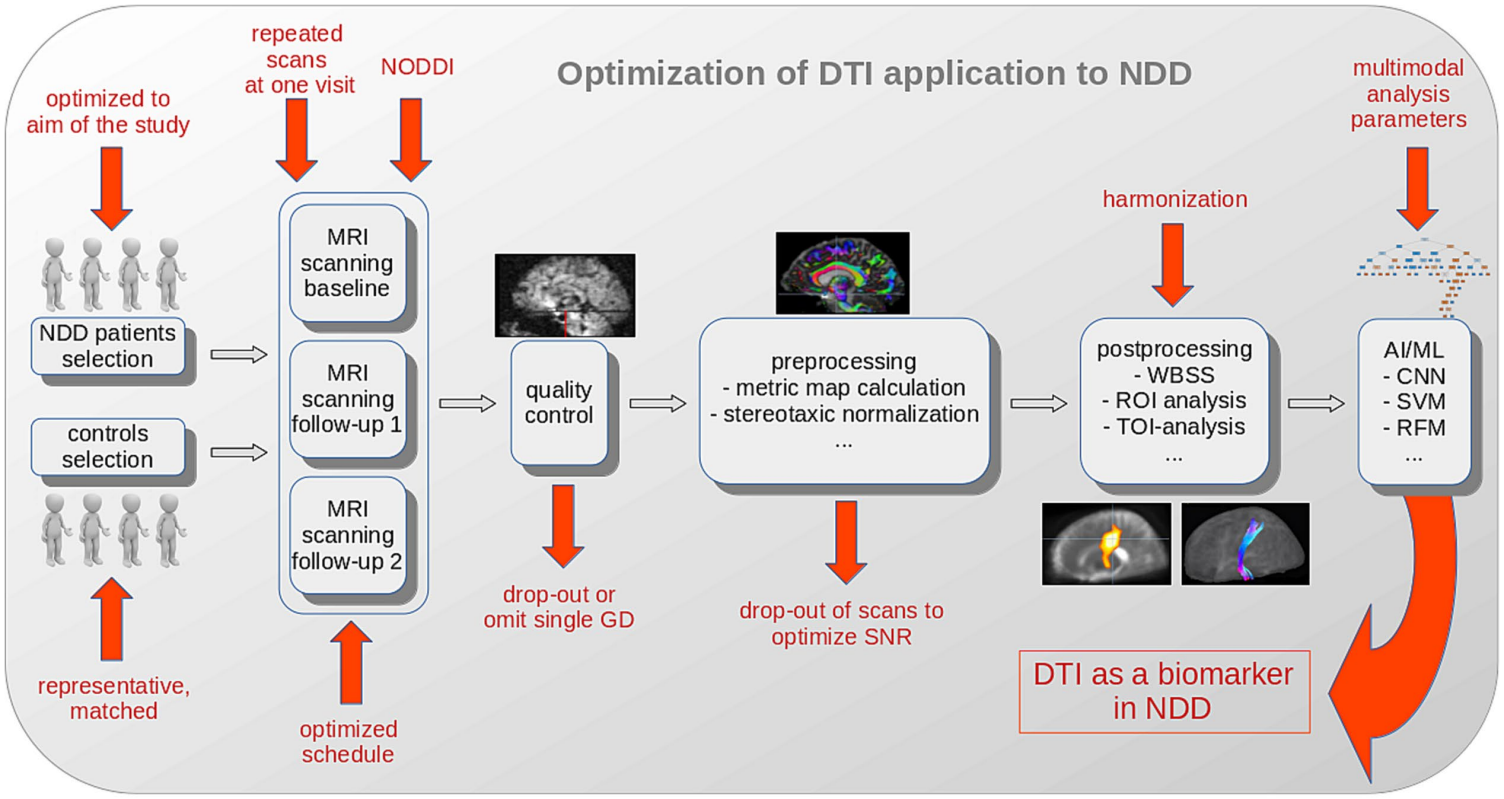
Schematic illustration of optimization options for DTI analysis to establish DTI as a biomarker in NDD. The selection of NDD patients should be optimized to the aim of the study and a representative and matched control sample should be collected. Extending the DTI protocol to a neurite orientation dispersion and density imaging (NODDI) sequence, the repetition of scans at one visit, and an optimized schedule substantially improve the results. Quality control and parameter comparison could lead to drop-outs with the goal to improve the SNR in a study. After (center-, scanner-or protocol-) harmonization, AI/ML methods (with inclusion of additional multimodal analysis parameters) potentially further improve the outcome in a given study. AI, artificial intelligence; CNN, convolutional neural network; FA, fractional anisotropy; GD, gradient direction; ML, machine learning; MRI, magnetic resonance imaging; NDD, neurodegenerative disease; RFM, random forest method; ROI, region of interest; SVM, support vector machine; TOI, tract of interest; WBSS, whole brain-based spatial statistics.

In addition to the hardware and software already tailored to optimize the SNR of individual recordings by the manufacturer, significant improvements can be achieved in the selection of study participants, the design of the studies, the application of the DTI protocols and the data analysis in pre-and postprocessing with regard to the effect size and thus the sample size in longitudinal clinical studies/trials (Figure 2).

This summary of recommendations was conducted to set up a framework for a standardized DTI scanning and DTI analysis protocol in studies in NDD (Figure 2):

- The healthy control sample measured at baseline should be representative, i.e., controls should be carefully selected (age-and gender-matched as well as similar level of education and living conditions) and there should be no hesitation in excluding measurements of poor quality from the control sample. Under the condition of outliers or a high variability in controls data, the reduction of the control sample (to a more representative one) leads to an increase in the effect size.
- Simulation studies showed that the statistical power of longitudinal DTI studies in NDD can be substantially increased by multiple scans of the same subject per session, especially in limited sample sizes. Such optimized study protocols can help to establish DTI metrics (like FA) as an imaging biomarker in NDD, especially to monitor disease progression in the natural history.
- For longitudinal studies in NDD, one baseline and two follow-up visits were advised (especially for diseases with a rapidly progressive disease course) with the individual schedule for NDD patients that the timing of follow-up 1 does not bisect the entire observation period.
- For longitudinal analysis, efforts should be spent to an as large as possible control data set at baseline to obtain higher effect sizes by comparison of data of each visit (of NDD patients) to a large and optimized control sample at baseline.
- A standardized DTI protocol might be extended by additional b-values of up to 2000 s/mm^2^ to enable NODDI (a multishell diffusion technique) to enable analysis of further types of microstructural environments. Analysis possibilities of NODDI have been demonstrated to provide additional value for evaluation of NDD (Andica et al., 2020).
- The inclusion of ML methods in the analysis cascade allows for improved DTI-based analysis of WM integrity and its affectations by NDD (also in a multimodal setting).

For neuroimaging studies as a part of clinical therapeutic trials in NDD, general clinical recommendations should be considered: first, to obtain a homogeneous study population, i.e., the disease progression rate and also the genetic phenotyping of NDD patients should be part of the inclusion/exclusion criteria. Second, primary endpoint and treatment duration must be matched to the study population, especially for slowly progressive NDD patients.

### 4.2 Effects of data collection on results quality

The potential of DTI-based metrics as a non-invasive progression marker during the disease progression in NDD is influenced by sample size, scheduling of baseline and follow-up sessions, and measurement uncertainty on the statistical power. Especially for measurements with a limited SNR, for example, due to subject-related factors (Müller et al., 2013), the application of the recommendations of this review will potentially strengthen the reliability of the FA values, in line with SNR improvement by signal-averaging during individual scans (Farrell et al., 2007; Seo et al., 2019). Vice versa, the increased statistical power of a DTI protocol means that lower sample sizes might suffice to measure small effects and/or effects after a short time, respectively (Behler et al., 2022a). Another critical factor is the composition of the groups and the selection of the scans to be included in the analysis. Prior to analysis, the restriction to carefully selected patients and controls saves costs and could increase effect sizes, that way also reducing patient burden and increasing subject motivation. It has to be added that data collection (selection of participants) as well as data quality have to be optimized during the process of data acquisition because subsequent *ex post facto* correction for age, different study protocols and quality control of movements and other artifacts can only partially correct for such deviations and could, thus, never reach the data quality of optimized (standardized) recordings and subject selections.

The technical aspects of planning medical trials listed here can generally be implemented with little effort, but when implemented in full or in part, they have the potential for a significant improvement in results with a simultaneous potential reduction in costs.

### 4.3 DTI as a tool to distinguish between different NDD

Based on characteristic alteration patterns of cross-sectional comparison of NDD patients to controls, DTI appears to be useful at distinguishing frontotemporal dementia (FTD) from patients with AD, also at the individual level (Grossman, 2010). DTI has shown to be able to classify subjects diagnosed with PD, atypical parkinsonism, and essential tremor and to distinguish them from control subjects (Prodoehl et al., 2013). DTI indicators of white matter impairment have the potential to emerge as useful clinical tools for differentiating diagnostic groups in studies of AD, MCI, and normal aging (Nir et al., 2013, 2015). ALS and FTD encompass a clinical, pathological and genetic continuum, and ALS could be mainly distinguished from ALS with frontotemporal dementia (ALS-FTD) and behavioral variant FTD by different white matter microstructure alteration pattern, especially corticospinal tract degeneration (Lillo et al., 2012).

### 4.4 Application of machine learning

DTI has been used to study the effects of NDD on neural pathways which may lead to more reliable and early diagnosis of these diseases as well as a better understanding of how they affect the brain. In AD, ML methods were applied for defining DTI metrics (Konukoglu et al., 2016; Lombardi et al., 2020; Xu et al., 2021; Agostinho et al., 2022) to characterize MCI (Velazquez and Lee, 2022; Zhou et al., 2022a,b; Cheng et al., 2023) and to predict AD early (Savarraj et al., 2022). The characterization of MCI and cognitive impairment in PD (Xu et al., 2021; Yang Y. et al., 2022; Chen B. et al., 2023; Huang et al., 2023) or the investigation of progression in PD (Prasuhn et al., 2020; Yang et al., 2021a,b) has also been addressed by the application of ML methods. Furthermore, ML was applied to the differentiation of parkinsonian syndromes (Haller et al., 2012; Du et al., 2017; Chougar et al., 2021; Talai et al., 2021). Since first applications of ML methods to ALS (Welsh et al., 2013; Sarica et al., 2017), ML was used to improve diagnostic accuracy (Kocar et al., 2021; Behler et al., 2023) and clinical associations (Li et al., 2021). For the evaluation of imaging biomarkers in HD, ML methods have been applied for many years (Klöppel et al., 2008; Rizk-Jackson et al., 2011; Hu B. et al., 2023). Thus, ML and AI are exponentially improving medical imaging and diagnosis: by ML techniques (especially SVM or RFM), the combination of multiparametric/multimodal imaging data allow for a multidimensional analysis beyond (simple) association analysis (Tae et al., 2018; Weston et al., 2023; Takahashi et al., 2024). Moreover, CNNs might enable estimations of the risk status, and thus, prediction of the disease course including survival in NDDs (Agosta et al., 2019; Kuan et al., 2023). Altogether, it seems safe to expect that ML methods will help to define the future of DTI-based analysis of WM integrity in the brain and its affectations by NDD.

### 4.5 Consideration and limitations of the DTI in neurodegenerative diseases

Technical considerations are presented that improve the information contents in DTI recordings and subsequent statistical analysis in clinical DTI-based neuroimaging trials in NDD. In summary, this study and the included recommendations potentially might enhance the role of DTI as a biomarker in NDD by standardized scan protocols and analysis cascades.

Especially AI-based techniques improved the performance of multimodal MRI including DTI in survival prediction and the prognostic value in NDD (Agosta et al., 2019). Furthermore, AI-aided DTI analysis provides high-precision automatic diagnosis and simultaneously output feature probability maps to provide clinical auxiliary diagnosis in NDD (Deng et al., 2023). Thus, there is the urgent need for the translation of advanced brain MRI techniques into clinical practice, e.g., for an assessment of prognostic factors and a stratification of patients in the design of pharmacological trials (Agosta et al., 2019). That way, the predictive value of microstructural integrity by AI-based algorithms, especially in a multiparametric or multimodal setting, could be developed as a non-invasive *in vivo* biomarker in NDD.

## Author contributions

H-PM: Conceptualization, Formal analysis, Methodology, Visualization, Writing – original draft, Writing – review & editing. JK: Conceptualization, Formal analysis, Methodology, Visualization, Writing – original draft, Writing – review & editing.
